# The Tug of War

**Published:** 1886-03

**Authors:** 


DANIEL'S

Medical ★ Journal,

PUBLISHED MONTHLY AT

AUSTIN, TEXAS.

F. E. DANIEL, M. D., EDITOR AND PUBLISHER

Subscription Price: Two Dollars per Annum in Advance.

Papers for publication in this Journal should be in the hands of the Editor by the 10th of the month preceding the month in which it is wished to appear; they must be original,—never having appeared in print elsewhere, and must be contributed exclulsively to this Journal.

Contributions to the department of Original Articles are cordially invited from the professiom of Texas, expecially. What is most desired is Clinical reports of cases, essays and short practical articles on any medical topic; also solicited reports of proceedings of societies, clubs, etc., and items of medical news of general interest to the profession, such as notices of appointments of physicians, removals, changes, marriages, deaths, etc.

### EDITORIAL.

### THE TUG OF WAR.

"We but catch at the skirts of the thing we would be,
And fall back on the lap of a false destiny."
—OWEN MEREDITH.

It is generally believed a desperate battle is to be fought in the Medical Convention at St. Louis in May. The American Medical Association and the Code of Medical Ethics are to be assaulted by an overwhelming force of wily and desperate men, especially packed for the occasion; the same who, allied with the new code men, at New Orleans, attempted to capture and run away with the Association, and failing, called those who defea ed their plot, "plotters and schemers," etc., and accused them of doing just what they failed to do. These gentlemen, mostly city specialists, allied to the new code men and a few army aristocrats, finding the Code of Ethics interfere with the exercise of their money-making schemes—chief amongst which is consultation with any, and everybody, who will bring a fee, would do away with it. Hence the war on the only national medical organization in America. The demand for admission of new code men into the International Medical Congress through the doors of the American Medical Association, is only a pretext for this assault. They know that their demand, their ultimatum that the American Medical Association shall rescind its action had at New Orleans with reference to the organization of the Congress, and the restoration of the original committee to the *status quo ante* New Orleans, is preposterous, and will not be listened to; they know that the Association cannot honorably, and, therefore, will not, recede from the position taken; and that is just what they want That will, then, they think, give them the pretext for the assault. Why, we ask, are these gents so concerned for the admission of new code men—men who have voluntarily withdrawn from affiliation with the regular profession, into the Congress as delegates from the Association which they have left? Is it because Dr. Shoemaker *told he truth*, and they are *honor bound* to make good their promises to Jacobi and company, made at Copenhagen, that if they —J. & Co—*would not defeat the acceptance of the American Medical Association's invitation to the Congress to meet in America, they would see that Jacobi and his new code friends were appointed to office in the Congress?* or is the admittance or refusal of these men a mere pretext, as we have said, to make war to the knife on the code, the only restraining influence they recognize, in the temptation to consult with quacks?

We are not advised as to the mode of attack contemplated; but the recent capture of the Philadelphia County Medical Society, whereby delegates in the interest of this plot were made to set aside the regular constitutional nominees, will give us some insight into the tactics they will employ; and which the South and West will be called on to defeat—a second time.

The necessity that the South and West should send their very best men to the front—charged to stand by the Association in the assertion of its rights, and who *are loyal to the code*, is, therefore, apparent. At New Orleans, the Texas delegation, the largest on the ground, stood by the decision as a man. They were not prepared to admit that the profession of medicine in America is resident solely in New York, Philadelphia and Boston; and could not quietly submit to having the Empire of Texas left entirely out in the cold, and without representation in the Congress, seeing she is so largely and so well represented in the National Association. This time, the Texans will be called on to, not only stand by the Association, but to do some fighting in its behalf, we opine. Hence we exhort our County Medical Associations, and our State Association to send as delegates, only such men as are not *afraid to speak out* in defense of the right. God is with the right. Our cause is just, and must prevail. We must defeat the medical communists at St. Louis, or forever bid adieu to honorable medicine in America. These *soi disant* medical "leaders" have staked their all on the die, and will lose. They have clutched at the skirts of medical autocracy—would-be dictators, and have fallen back on the lap of a (just) destiny. They have illustrated Shakespeare's "vaulting ambition" o'erleaping itself. They have treated the American public to a leap of unprecedented elasticity and stretch, and missing their mark, and have fallen into a quagmire from which all the sophistry of the *New York Medical Record* will never be able to extricate them. After the St. Louis Convention, they can face Shrady & Co., and, in the language of the Mikado, exclaim: "Here's a pretty how d'ye do,"

